# Supplementary material for: Identification of photoperiod-induced specific miRNAs in the adrenal glands of Sunite sheep (Ovis aries)
Source: Front Vet Sci. 2022 Jul 22;9:888207. doi: 10.3389/fvets.2022.888207 (PMC9354845; doi:10.3389/fvets.2022.888207)
Supplement: Supplementary file 1 [file Data_Sheet_1.ZIP › Supplementary file/miRDeep2 file.zip!/miRDeep2 file/Novel_Structure/LP42A1/Novel_164.pdf]

Provisional ID	:	NC_019469.2_20605
Score total	:	9.1
Score for star read(s)	:	3.9
Score for read counts	:	1.7
Score for mfe	:	1.9
Score for randfold	:	1.6
Score for cons. seed	:	
Total read count	:	15
Mature read count	:	14
Loop read count	:	0
Star read count	:	1

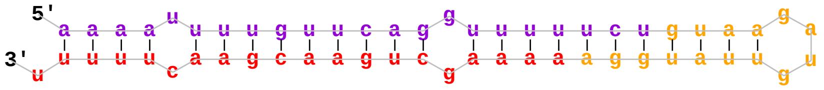

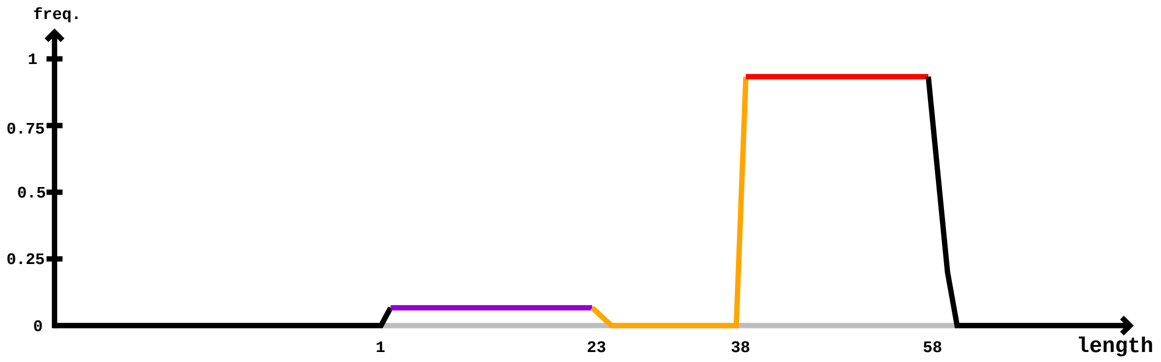

Star Mature

```
5'- ucuuuaggguuuucuuuauguuggguuggcaaaaaaauuuuguucagguuuuucuguaagauguuauggaaaaagcugaacgaacuuuuuggccaacccaguaguaucau   -3'    obs
    ucuuuaggguuuucuuuauguuggguuggcaaaaaaauuuuguucagguuuuucuguaagauguuauggaaaaagcugaacgaacuuuuuggccaacccaguaguaucau          exp
    ....(((((....)))))..((((((((((..(((((.(((((((((.(((((((((((....))))))))))).))))))))).))))).))))))))))......... reads   mm   sample
    .................................aaaaGuuuguucagguuuuucu....................................................... 1       1    seq
    ......................................................................aaaaUcugaacgaacuuuuu.................... 8       1    seq
    ......................................................................aaaagcugaacgaacuuuuu.................... 1       0    seq
    ......................................................................aaaaCcugaacgaacuuuuug................... 2       1    seq
    ......................................................................aaaaUcugaacgaacuuuuugg.................. 2       1    seq
    ......................................................................aaaagcugaacgaacuuuuugg.................. 1       0    seq
```
